# Establishing trust in HIV/HCV research among people who inject drugs (PWID): Insights from empirical research

**DOI:** 10.1371/journal.pone.0208410

**Published:** 2018-12-05

**Authors:** Roberto Abadie, Shira Goldenberg, Melissa Welch-Lazoritz, Celia B. Fisher

**Affiliations:** 1 Department of Sociology, University of Nebraska-Lincoln, Lincoln, NE, United States of America; 2 Faculty of Health Sciences, Simon Fraser University, Bumaby, BC, Canada; 3 Pharmaceutical and Nutrition Care, University of Nebraska Medical Center, Omaha, NE, United States of America; 4 Department of Psychology, Fordham University, Bronx, NY, United States of America; Centers for Disease Control and Prevention, UNITED STATES

## Abstract

**Background:**

The establishment of trust between researchers and participants is critical to advance HIV and HCV prevention particularly among people who inject drugs (PWID) and other marginalized populations, yet empirical research on how to establish and maintain trust in the course of community health research is lacking. This paper documents ideas about trust between research participants and researchers amongst a sub-sample of PWID who were enrolled in a large, multi-year community health study of social networks and HIV/HCV risk that was recently conducted in rural Puerto Rico.

**Methods:**

Qualitative research was nested within a multi-year *Social Network and HIV/HCV Risk* study involving N = 360 PWID > 18 years of age living in four small, rural Puerto Rican communities. Semi-structured interviews were conducted between March 2017 and April 2017 with a subset of 40 active PWID who had been enrolled in the parent study. Interview questions invited participants to draw upon their recent experience as research participants to better understand how PWID perceive and understand participant-researcher trust within the context of HIV/HCV-related epidemiological research.

**Results:**

Fear of police, stigma and concerns regarding confidentiality and anonymity were identified as structural factors that could compromise participation in HIV/HCV-related research for PWID. While monetary compensation was an important motivation, participants also valued the opportunity to learn about their HIV/HCV status. During their participation in the study, gaining knowledge of safe injection practices was perceived as a valuable benefit. Participant narratives suggested that PWID may adopt an incremental and ongoing approach in their assessment of the trustworthiness of researchers, continuously assessing the extent to which they trust the research staff throughout the course of the research. Trust was initially generated through peer Respondent Driven Sampling recruitment. Research staff who maintained a presence in the community for the entire duration of the prospective study reinforced trust between participants and the research team.

**Conclusion:**

Although PWID face numerous structural barriers to research-related trust in HIV/HCV research, we found that using a peer-based recruitment method like RDS, and employing a research staff who are knowledgeable about the targeted population, culturally sensitive to their needs, and who maintain a long-term presence in the community may help mitigate many of these barriers. The reputation of the research is built incrementally as participants join the study. This contributes to a “street reputation” that grows as current or former participants vouch for the study. Establishing trust was identified as only the first step towards building a collaborative relationship with participants, and our findings suggest that steps to address criminalization and stigmatization also are necessary to support research trust.

## Introduction

Trust in research plays a significant role in the recruitment and retention of vulnerable populations who are often excluded from community health research [1–5]. Barriers to the recruitment of PWID disrupt efforts to address HIV and HCV transmission among this population [6–9]. Research dishonesty and malpractice has played a role in shaping community responses to the research enterprise [10–14]. Numerous studies have shown that race significantly shapes attitudes towards trust in biomedical research, at least in the mainland US. African Americans, in particular, have been more distrustful of medical research, after Tuskegee and other past abuses [15–18]. Native Americans also share some of these same misgivings, and in particular, tribes have been very vocal in recent efforts like the Human Genome Diversity Project about the ethics of conducting research involving their communities which they perceive to be exploitative [19–21]. Other minority groups have been found to be severely under-represented, particularly in clinical trials research [22–24]. On the other hand, some authors have shown that systematically targeting the same ethnic groups for research can also lead to resentment and mistrust [25]. Such sentiments might be compounded in the case of PWID, which are often stigmatized and discriminated against [26–28].

Recruiting and retaining PWID is critical if we are going to successfully address the HIV/HCV epidemics. A number of studies about the effects of trust in accessing services among PWID exist. For example, a study by Treolar et al., conducted among PWID receiving services from a Service Exchange Provider in Australia, showed that stigma represents a significant barrier to trust but that a respectful attitude on the part of the staff recognizing the individuality of PWID can enhance trust in and their predisposition to receive health services [29]. Authors suggest that staff at the SNP viewed trust as a result of a deliberate effort to build and maintain trust through their interactions with PWID. Harris et al. reached a similar conclusion in their study of PWID seeking HCV treatment in the UK [30]. Another study about barriers to enrollment in methadone-assisted treatment among women in Tanzania showed that discrimination suffered by women who inject drugs leads to distrust, preventing them from enrolling in the program [31]. Stigma has also been identified as a barrier to accessing HIV clinical research and treatment among PWID [32,33]. As Meyerson et al. document in a study of HIV testing, there are many instances in the provision of this service where providers can enact or reinforce stigma among PWID [34]. We suggest that this might also be the case in the interactions between researchers and PWID.

A number of studies drawn from organizational behavior, health care settings and economics, among other fields, have shown that trust is a relational concept that may have varying meanings for different actors. According to these studies, trust is not a static concept; instead, trust is produced through shared cultural norms, expectations of reciprocity and institutional arrangements that shape social interactions [35–37]. Trust formation in health research shares some of these attributes, particularly, participants’ desire to contribute to the research goals in the context of a reciprocal arrangement in which both researchers and participants see themselves as partners in the research enterprise [38–42]. Our study data support these views. Our own findings from the study presented here, show that research participants were initially distrustful of research staff but through their engagement with the study their attitudes shifted, their concerns diminished, and their trust increased. Central to this process, was their perception of a reciprocal relationship. Participants saw themselves as contributing valuable data to the study, and saw researchers as supporting participants by providing fair payment and HIV/HCV test results in a supportive and confidential environment.

While these authors provide a valuable template to understand the social basis of research trust, this process is not well understood among those people who inject drugs who are enrolled in health research. To explore the barriers and facilitators to research trust among PWID, we conducted a study based on a sub-sample of participants who were previously enrolled in a large study on social networks and risk in Puerto Rico. Our aim is to document how trust is understood by this vulnerable population, and to describe the process of gaining trust, paying particular attention to the extent to which their participation was based on that trust or whether, despite distrust, they participated for other reasons. One of the main strengths of this study is that participants’ perceptions of research trust are based on their actual experiences of having participated in a large empirical study, as opposed to purely hypothetical scenarios about research trust.

Understanding the mechanisms by which research trust is established and maintained among this stigmatized population may improve recruitment and retention in epidemiological studies, contributing thus to the production of valuable social knowledge not only in the US but also in international settings. The most recent HIV surveillance data from the Centers for Disease Control and Prevention indicate that Puerto Rico has one of the highest incidences of HIV infection in the United States. In 2010, the island reported 28.2 new HIV infections per 100,000 residents, a rate over 1.5 times that of the US average, and the third highest of all the nation’s 56 states and dependent areas; yet, of the 103 major metropolitan areas, San Juan was ranked 20th in terms of new diagnoses of HIV in the same year [43]. A study of PWID in metropolitan San Juan showed a HIV prevalence of 17% and a 90% HCV prevalence, respectively [44]. In addition, a more recent study among rural PWID in Puerto Rico documented a HIV prevalence of 6% and a HCV 78.4% rate [45].

This issue is more pressing after Hurricane Maria devastated health services on the island, exposing an already vulnerable population to an increased risk of HIV/HCV transmission. Findings provide a participant-based perspective on trust, both to researchers and IRBs, facilitating evidence-based decision making while improving research fair selection and, ultimately, outcomes.

## Materials and methods

### Participants

In-depth interviews (n = 40) were conducted between in March and April 2017 among active PWID ≥ 18 year old males residing in the localities of Cidra, Cayey, Aguas Buenas and Comerio, in rural Puerto Rico. Our decision not to include women in the sample reflected the fact that women constituted a significant minority—around 10%—of PWID in rural PR [46]. This study was nested within an ongoing, multi-year NIH/NIDA-funded parent project on *Social Networks and HIV/HCV risk in Rural Puerto Rico* that was conducted between April 2015 and December 2016. The parent study is divided into three phases. As previously described [47], the first phase consisted of an epidemiological risk survey, HIV and HCV testing to assess sexual and injection risk behaviors of PWID residing in these municipalities, as well as their degree of access to health-promoting services. Phase one included N = 315 active PWID, also 18 ≥ years old, who were recruited using respondent-driven sampling (RDS), a well-established sampling methodology for recruiting hidden and hard-to-reach populations [48–50].

### Procedure

Using RDS, recruitment was initiated by starting two seeds in each of the four municipalities (for a total of eight seeds and 307 recruits). Participants in the first phase received $25 in compensation, and were also given the chance to become recruiters. After securing consent, they were provided with three referral coupons to recruit other PWID who had not previously participated in the study. Every eligible referral earned the recruiter an additional $10. For the second phase, we engaged in ethnographic research with 34 “Key Respondents” to learn more about injection risk practices and the structure of their social networks; this included visiting their homes, shooting galleries and other locations where they might inject. In this phase we included 45 participants who were part of the key respondents’ ego networks but who had not participated in the first phase of the study. Finally, for the third phase, we purposefully selected 24 participants from the previous two phases to participate in a weeklong training on safe injection methods. Participants in our trust study received $30 in cash and were handed pamphlets with information about how to prevent HIV/HCV transmission.

We secured approval from the University of Nebraska-Lincoln Institutional Review Board Committee on Human Research, for both the parent study and the present study on research trust, before we initiated data collection. All participants provided written consent at the study office prior to enrollment in the study. The informed consent process was informed by previous research with PWID in this context and the form was written in Spanish. A written copy of the consent form including contact numbers for the Principal Investigator. To ensure that participants had fully grasped the main information contained in the consent we included follow-up questions to assess comprehension, including probes such as: what is the aim of the study? What are we asking you to do? To ensure confidentiality, participants were identified through a unique ID code, which also facilitated the linking of participant data to data gathered through the parent study (e.g., demographics, HIV/HCV status, polysubstance and injection drug use, and injection risk behaviors). A Community Advisory Board (CAB) of eight active PWID who had participated in the parent study was established prior to commencing data collection. Input of the CAB was sought in drafting the research design, as well as the recruitment and consent procedures, to ensure their cultural appropriateness and sensitivity to our study population. CAB members met twice, once at the beginning of the study and once the study was completed to discuss research findings.

### Interview format

In-depth interviews were conducted by the PI who had extensive experience not only in this technique but also in working with the same population. With the permission of participants, all interviews were audiotaped at the research office site in Cidra, a place already familiar to study participants. The first portion of the in-depth interviews collected demographic data, such as age, education, income, costs of acquiring drugs, frequency of drug use and access to health care, which together complemented the broader demographic information, risk profiles, and HIV/HCV status data available through the parent study.

The main focus of the interviews was to document emic understandings of trust in research and assess the potential barriers and facilitators of trust in community-based, epidemiological studies involving PWID. A semi-structured questionnaire was used to explore participants’ views on this topic. The participants were asked about their motivations to enroll in the parent study as well as their understanding of its aims, risks and benefits. Participants were asked if they had any initial misgivings about enrolling and, if they did, why and how they overcame them. To assess the effect of RDS we asked participants how the experience of being recruited by another drug user who was compensated for their participation affected their disposition to participate. A probe such as “Did you trust our study because it had been introduced by another user that vouched for us?” was used to elicit views about this topic. In addition, we probed participants’ attitudes about the researchers’ obligations towards participants and their own obligations towards research participation. The interviewers asked participants directly what would lead them to trust a new researcher coming to the area and what kinds of information (if any) they would be reluctant to reveal to a new investigator.

### Data analysis

In-depth interviews were transcribed and all personal identifiers were removed. The qualitative software MAXQDA was used to manage coding. Data analysis was conducted by the PI. Codes were developed to convey the wide range of themes present in the narratives of PWID. An audit trail was maintained to keep track of how and why analytic decisions were made, and a codebook was developed to describe and define all study codes. As it is practiced in qualitative analysis, these codes were iteratively revised and regrouped until they eventually represented a set of higher-level axial codes comprehensively describing participants’ perceptions regarding the emic meanings of trust, its barriers and facilitators. Following Strauss’s grounded theory approach [51], the interpretation of the data emerged inductively from the data, instead of imposing a pre-existing theoretical framework to fit the data. All names reported in the results are pseudonyms.

## Results

### Participants’ characteristics

The median age of the participants was 42.4 years [Table 1] and the median number of years spent injecting drugs was 22.5. Almost one-quarter were HIV-seropositive and a large majority were HCV-seropositive. Of particular concern, all participants reported having previously experienced incarceration. Almost all participants were unemployed, and approximately half had never been married and had a high school or greater level of educational attainment. Finally, a little more than half injected four times or more a day and one-third injected two to three times a day. Few reported having participated in a research study other than the parent study from which they had been recruited.

**Table 1:** Characteristics of participating male people who inject drugs (N = 40), rural Puerto Rico, 2017.

~	n	% / mean	Range
**Male**	40	100%	
**Age**	40	42.4 years	25–59
**# of Years Spent Injecting Drugs**	40	22.5 years	2–41
**HIV+ Status**	2	5%	
**HVC+ Status**	33	82.5%	
**Previous Incarceration**	40	100%	
**Previous Research Experience**	3	7.5%	
**Marital Status**			
**Married/Living Together As Married**	7	17.5%	
**Separated/Divorced**	7	17.5%	
**Widowed**	3	7.5%	
**Single/Never Married**	23	57.5%	
**Highest Level of Education**			
**Grades 1–6**	1	2.5%	
**Grades 7–9**	11	27.5%	
**Grades 10–11**	8	20%	
**Grade12/GED**	15	37.5%	
**Some College**	5	12.5%	
**Employment Status**			
**Employed Full Time**	3	7.5%	
**Employed Part Time**	1	2.5%	
**Retired**	1	2.5%	
**Unemployed**	35	87.5%	
**Frequency of Injection**			
**4 or more times per day**	22	55%	
**2–3 times per day**	12	30%	
**One time per day**	1	2.5%	
2–6 times per week	4	10%	
2–3 times per month	1	2.5%	

### Qualitative research findings

#### “Are you a cop?” Fears regarding research enrollment among PWID

Since all interviewees had experienced prior incarceration–usually for non-violent, drug possession charges–most initially had feared that researchers were either undercover cops or “snitches”. Intense police crackdowns on drug-selling locations in Puerto Rico affect PWID, who can be arrested acquiring the drugs or working as “watchers” alerting drug sellers to the presence of cops in the area: “When we first saw you, we didn’t know who you were and we suspected you could be an undercover cop, or a snitch. It has happened many times before that they [cops] infiltrate the area to get information before making their operations” (Foca). Yet, for most PWID it didn’t take long to realize that our research team had nothing to do with police: “I can recognize a cop a mile away. I have been so many times in jail that I developed a sixth sense, I always recognize an undercover cop. And besides, just the way you look, the way you talk, the education, everything is very different from a cop” (Walter). Participants in our study did not fear retaliation from drug dealers if they engaged in our research, and some even argued that the fact that they enrolled in our study and got paid for their participation was “good for the dealers” because participants would then spend their earnings acquiring drugs.

#### Trust in other PWID recruiters

A more serious barrier to participation is stigma. Having been stigmatized as drug users throughout their lives, some were fearful of being discriminated against by research staff. In addition to the stigma of being a drug user, participants have to deal with the compounded stigma of being poor and having experienced a criminal conviction. While initially participants had misgivings about enrolling in the study, fearing they could be stigmatized again, this concern was quickly alleviated, initially by their peers who had already volunteered in the study and told them that this was not the case, and later, through their own experience when they visited our office and felt that they had been treated “with respect”. Our sampling method ensured that we didn’t directly approach prospective participants but that, instead, participants were recruited by other members. If prospective participants had any misgivings or concerns about participating, they could ask questions of the person inviting them to the study and giving them the coupon. All participants in our study stated they had done this and that it reassured them since the information came from somebody they knew and trusted: “The person that gave me the coupon told me about the study, what you were doing, and the tests, how long did it take and about the money. I have known him for years and I trust him, so when he told me about it, I thought, why not?” (Oski).

#### Confidentiality matters

The parent study did not ask participants for any private information. Unlike eligibility procedures for social services that demand a picture ID as form of identification as well as a social security card, we only identified participants by a code ensuring that their anonymity and confidentiality would be maintained. Some participants expressed some initial misgivings about privacy of their personal information, expressing doubts about how it would be stored and used. “At the beginning I was a bit scared because I didn’t know if this study was for Puerto Rico or for another country. Here in Puerto Rico I have my daughters and they don’t know their dad is an addict. I didn’t want to have my picture in a magazine, or in TV and [where] my daughters can see me” (Chino).

The possibility that HIV/HCV test results could be leaked in a small rural community was an important consideration for most participants: “I had a concern about how private the test results would be, I wouldn’t like that my face appears on the internet with somebody saying, ‘this guy has HCV’ and my photo shows up! I like my privacy but I have no problem participating in a medical study if my privacy is maintained” (Cesar).

While a large majority of participants had not been involved in research prior to the parent study, most had regular access to medical care and were well aware of HIPAA, the regulations that protect the privacy and confidentiality of their medical records. Participants assumed that HIPAA or a similar arrangement would protect their confidentiality and privacy as research subjects and had no problem in understanding the assurances we provided during the informed consent process: “If you don’t have that requirement [confidentiality] you would not be here wasting your time to deal with us. It is a requirement that you have in order to do this study with us. Without it, all your work would be useless” (Pablito).

In addition, participants sought to confirm during their visit to our research office that no private information would be requested and that anonymity would be maintained: “Yes, I am reassured because I can see that you are not using my full name, or my social security number, or anything else. I always had wanted to come [to the study]. I am at ease because nothing comes out of this office” (Landi).

#### “Money always helps”: Motivations and perceived benefits of research participation

Financial gain, in particular for those PWID who had a high frequency of drug injection and thus needed significant financial resources, was an important motivation to participate. In addition, many participants expressed their desire to know their HIV/HCV status. While most participants had been tested in institutions like the prison or drug treatment programs, those tests may have taken place some time ago. In addition, institutional testing is conducted under a public health approach that mandates disclosure of prior sexual and injection partners, among other requirements, in case of a positive result. That our study was not subjected to these same reporting obligations was seen by participants as a valuable opportunity to receive an up-to-date assessment of their HIV/HCV serostatus. “Money always helps, but I also came in for the tests, because I have been doing things, taking risks and I wanted to know if I had it [HIV] or not” (Carlos). Gaining knowledge of their HIV status was perceived as an opportunity to proactively take care of their own health: “I thought that I could get HIV tested and I could know if I had it or not. I thought, I get checked and with this I have nothing to lose, on the contrary, I win because if I have any health condition like HIV I can find out” (Miguel).

In addition to HIV/HCV testing we provided counseling influenced by harm reduction practices, informing participants in a nonjudgmental way of safe ways to inject. Participants perceived this as a benefit of enrollment: “All the advice we got about not sharing the same syringes to avoid diseases” (Canito). Having a conversation with a trained staff member about safer injection practices had, for some participants, an additional effect, leading them to less their drug injecting frequency in order to limit risk exposure: “I learned things that helped me take care of my health and also this motivated me to cut my drug use since the first time I came to the study, I am not using now as much as I was using before” (Cieguito). For many participants dealing with stigma, and fighting loneliness or other mental health issues, finding a safe space where they could get a respite from the hustle associated with the drug life was perceived as another benefit of participating in the research. “The way you treat us, with respect, like we want to be treated. We can come here, get cookies, juice, and just talk without fear of been looked down, or judged for what we are” (Viejo de la Planta).

Finally, without previous exposure to a research study, a minority stated they had initially mistakenly assumed that we provided services, like syringe exchange, or would connect them to housing services or treatment options; this belief also motivated them to participate in the study. However, once they learned this was not the case, they still decided to enroll.

#### The process of establishing and maintaining research-related trust

Through their interactions with research staff, participants were able to assess whether staff could be trusted or not. They sought to judge whether we looked like cops or snitches, but also if the things that their peers had told them about confidentiality and anonymity were true. And “having been discriminated [against] all their lives” they were also looking for any sign of potential stigma or discrimination towards them. In so doing, they exhibited a pragmatic rationality, grounding their assessment first on what their peers told them and then using their own experience in order to make a judgement. Many participants expressed that it was important for them to “see for themselves” in order to form an opinion about the project.

The degree of their trust in the researchers did not change if participants later discovered that some of the expectations they initially might have held, such as greater access to services or even medical treatment for HIV or HCV, would not be met by our study. Participants admitted that “it was their fault” that they got HCV in the first place, not ours, and that they were aware that the necessary medication is not covered by Medicaid in Puerto Rico and that this was not a responsibility of the researchers. With regards to other services such as access to methadone or suboxone treatment, or assistance in securing housing arrangements, participants declared that they would “just look elsewhere” and again did not continue to expect this from the research team. Trust in the research and researchers did not seem to be affected by the degree of knowledge participants had over the goals, risks and benefits of our study. While all interviewed participants showed a high degree of trust, some had a more specific understanding of the parent study’s aims, whereas others could only recall very general goals such as “to improve our situation” or “test us to see how we are”, while a minority admitted they were unsure what the study goal was.

Even though some participated only in one phase and others completed all phases, a factor that seemed to influence their trust in our study is that they “could see us all the time”. Our team spent two and a half years in the area conducting research and that was reassuring for some of them: “I believed in your project because I saw what you were doing. It wasn’t the case that I saw you once and then never again. But, no, you were always there for us, helping us. Little by little, I got to know you. You stayed and people could see you all the time. If you see a group and then you don’t see them anymore, that would give me pause because I don’t know what you are going to do with that [research data]” (Bebo). Continuous and sustained research presence, either through participants’ visits to our office or during our routes with the study van, provided opportunities for interaction, and through these interactions participants got to “know us better” and their trust was not only maintained but was increased over time. PWID in the study saw their participation as a relationship, a form of reciprocity where “you help us and we help you”. Respecting their anonymity and confidentiality was one of the obligations they thought researchers had to maintain, and in turn, participants felt an obligation to “tell the truth and to assist in the project so you can have the results you need” (Manuel). This sense of obligation came from their understanding of the relationship that was built between researchers and participants: “there is nothing that prevents me from getting up and walking off right now”, one participant observed.

Our findings suggest that the trust participants developed in the parent study’s research staff was project-specific and not transferrable to another research team, indicating that a new team coming to the area to conduct research with PWID would have to “start all over again”. As they had done with our research project, they would assess whether to trust a new research team following the same blueprint: first, they would get information from peers, then rely on their personal experience. This process might take some time since, according to participants, instead of assuming research trust, members of the research team have to engage in a process of trust building: “it has to be little by little and they have to earn it” (Chanfle).

## Discussion

This study documented the social basis of trust in research among active PWID in rural Puerto Rico. Results show that participants adopt a highly pragmatic approach, relying on peer experiences with the research team as well as their own involvement in research in order to determine whether they should trust the research project.

Participants seem to adopt an incremental approach to research trust. First, being recruited by current participants provides them with the opportunity to ask questions about the study of their peers and to form a first impression about whether they wish to participate in the study. Second, once they are in the possession of a coupon, coming to our office for the initial eligibility screening provides them with an opportunity to judge for themselves whether the information they already have about the study is confirmed by their direct experience.

Establishing and maintaining trust seems to be a social process that should be sustained between research participants and the research team. Trust founded in reciprocity between participant and researcher, developed over time, also corresponds to researchers’ understandings of research trust [38]. This is not surprising since ethicists have previously argued that research participants are often able to engage in complex moral reasoning [52–56]. Our research is consistent with mainland studies indicating PWID engaged in or invited to participate in research adopt a pragmatic assessment of the research project and its staff in order to decide whether to trust them or not.

While research mistrust has been extensively documented among ethnic minorities, in particular in the US, [57–63] our research team did not have this experience.

Fear of police was extensive in this population and it is based on the punitive effects of the ‘war on drugs’ that has resulted in extremely high rates of incarceration for minor quantities of drug possession or other nonviolent crimes [64]. The same mistrust in police can be found among other vulnerable populations such as sex workers and can be an initial barrier to their participation in research. Yet, our study shows that this barrier can be overcome if participants perceive the study will benefit them or their communities. Our finding, that previous experiences with stigma and discrimination can also operate as barriers to recruitment, echoes similar findings with similar populations elsewhere [7,65–67].

Participants perceived many benefits in research participation. HIV/HCV testing enabled them to monitor their health status, enabling a more proactive attitude towards their care. This finding is consistent with studies of HIV patients enrolled in clinical trial research showing that participating in research empowered research subjects [68], as well as with research with other marginalized groups, including sex workers, who also perceive health-related monitoring to be an important benefit and motivator for research engagement [69,70]. Study participants also felt they benefited from their participation in other ways. The program’s emphasis on harm reduction strategies made participants feel welcomed into a space where they would not be judged or stigmatized while they received evidence-based recommendations for safe injecting. Participants appreciated this interaction and for some, the study was a motivation to reduce their drug injection frequency or to adopt of safer injection practices. This outcome is not unusual in the context of research studies involving PWID [71].

A trusting relationship cannot be established without a shared agreement among PWID that the research enterprise is something that will bring some kind of public good to them or their communities. Establishing trust requires that the research team earn and maintain “street credibility” or reputation among the research population. Prospective researchers aiming to conduct community health studies with PWID might avoid initial pitfalls in establishing trust by forming a local community advisory committee and/or consulting with community- or peer-based organizations serving PWID [72,73]. In addition, a culturally competent staff can greatly reduce the possibilities of misunderstandings that can arise in the researcher-participant relationship. Goldenberg et al. call attention to the particularities of local contexts, suggesting that research teams should take these factors into consideration while conducting research [74].

RDS proved to be a very effective tool in helping establish trust, at least initially in research among our population. Yet, other community research projects can equally benefit from the positive rapport and experiences of those participants who have been already enrolled in a study, if they are willing to endorse it. Establishing and maintaining trust is particularly important in longitudinal or long-term studies that require multiple visits or engagements of the research participants. A lack of trust might jeopardize ongoing data collection if participants suddenly decide to withdraw from the study.

Trust in research is critical to enhance the participation of marginalized populations like PWID, and eliminating barriers to their participation not only contributes to the production of important scientific knowledge but also can result in the implementation of prevention or treatment strategies that target this same group.

## Strengths and limitations

This study has some limitations that arise from the composition of our sample. Since all participants had already been enrolled in a larger community health study among PWID in rural Puerto Rico, we did not gather the perspectives of those that had refused participation in the parent study. Relying on the experiences of those participants that agreed to take part in our study prevented us from understanding the views of those that refused to participate. However, since the focus of our study was not on barriers to participation but on how trust is established and maintained in a research project, this limitation does not compromise the integrity of the study. Another limitation is that our sample only included men who were currently using intravenous drugs. Future studies aimed to assessing how women understand research trust in the context of community health studies among PWID should be conducted. Despite these limitations, a major strength of this study is that, contrary to a plurality of studies that inquire about ethical conduct of research, our study is not based on hypothetical situations formulated to elicit participants’ views but on participants’ actual research experiences. We believe that this choice enhances the validity of this study.

## Conclusion

In this study, we found that fear of police, absence of financial compensation, stigma and lack of confidentiality or anonymity can operate as barriers to research participation. Establishing and maintaining research trust can be enhanced by a recruiting method based on peer recruitment like RDS, and a research staff knowledgeable about the targeted population and culturally sensitive to their needs. Participants’ trust in research is built incrementally as participants develop more direct experience with the research. This contributes to the researchers’ developing a good “street reputation”, as participants vouch for the study to prospective participants. Establishing trust is only the first step towards establishing a collaborative relationship with participants, and maintaining trust is relevant particularly in longitudinal studies that involve repeated visits over time.
